# The genetic diversity and population structure of wild and cultivated *Avena* species in Ethiopia using a SSR markers

**DOI:** 10.1016/j.heliyon.2024.e38942

**Published:** 2024-10-22

**Authors:** Ashenafi Alemu Tiruneh, Kassahun Tesfaye Geletu, Nasser k Yao, Kifle Dagne Weldegiorgis

**Affiliations:** a Department of Biology, College of Natural and Computational Sciences, University of Gondar, Gondar, Ethiopia; b Department of Microbial, Cellular and Molecular Biology, College of Natural and Life Sciences, Addis Ababa University, Addis Ababa, Ethiopia; c ILRI-BecA Hub, Nairobi, Kenya; d Ethiopian Biotechnology Institute, Addis Ababa, Ethiopia

**Keywords:** *Avena* sativa, Genetic diversity, Simple sequence repeat (SSR) marker, Wild oats.

## Abstract

Oats are grains that can be consumed by both animals and humans. They have thrived in Ethiopia, where certain oat species are considered native to the region. This work represents the first investigation of the population structure and genetic diversity of Ethiopian and other country oats. This led the scientists to explore the genetic diversity and population structure of wild and cultivated Ethiopian oats (Avena) species as well as oat cultivated in USA, the Netherlands and Austria. This study's main objective looks to be to investigate the variation in genetic makeup of cultivated and wild oat species. Studying the population structure of the oat species in the germplasm of Ethiopia, USA, the Netherlands and Austria. We used nineteen fluorescent SSR (simple sequence repeat) markers since previous research had indicated that these markers had high PIC (polymorphism information content) values. Five species of Avena were studied among the 176 oat accessions: A. sativa (cultivated oats) and four wild oats, such as A. abyssinica, A. vaviloviana, A. fatua, and A. sterilis. The AMOVA investigation revealed significant genetic distinctions among populations, individuals, and within individuals, explaining 18 % of the variance within populations, 4 % among populations, and 78 % within individuals. The AMOVA analysis of Avena species demonstrated extensive variance, with 33 % variation among species and 67 % within each species, underscoring robust species differentiation. The study also discovered gene interchange between wild oat and cultivated populations, defining two Avena species: domesticated oats and wild oats. Using the STRUCTURE software at K = 2, PCoA, and UPGMA, a distinct genetic structure was displayed in the dataset. Despite variations in ploidy levels and genomes, A. sterilis and A. vaviloviana were determined to be more closely linked, whereas A. abyssinica and A. fatua demonstrated a close association. This research delivers valuable insights for scientists and can be employed in oat breeding programs to improve future oat yield and productivity.

## 1 Introduction

The origin of the cereal grain genus *Avena*, which belongs to the Poaceae family, is unknown, although it most likely originated in the Middle East or Mediterranean region [1]. Oat, primarily originating in the western Mediterranean, is cultivated in Asia Minor and spread as a weed in Europe [2,3]. Domestication occurred around 4000 years ago in northern Europe. Chawade [4], recognized around 32 species of oat, of which some are cultivated, such as *Avena sativa* L., but the others, which includes *A. sterilis*, *A. fatua*, *A. vaviloviana*, and *A. abyssinica*, are wild [[5], [6], [7], [8], [9]]. The genus has a basic chromosomal number of seven and is diploid, tetraploid, or hexaploid in ploidy.

Cultivated oats (*A. sativa* L.), an important cereal crop, come in sixth place overall in cereal production, behind barley, wheat, maize, and sorghum [10,11]. They feed people and animals, providing critical supplements [12,13]. *Avena* species, including *A. sativa*, are found in mild climates, with *A. sterilis* in Eurasia and northern Africa. Oats are a fast-growing, palatable, and nutritive fodder [14,15].

The tetraploid *A. abyssinica* and *A. vaviloviana* had the AABB genome [16]. Both cultivated *A. sativa* (common oats) and wild oats (*A. fatua* and *A. sterilis*) have the same AACCDD genomic composition [17]. The cultivated form of oats, which has a hexaploid genome, is closely linked to wild oats, which may supply useful genes for oat breeding [18]. Commonly, polyploid plants exhibit notable increases in biomass output, vigor, and resistance to environmental changes, which subsequently results in the development of essential agronomic traits in food crops [19,20]. and insect resistance, are present in wild oats and can have a direct or indirect impact on crop output and quality Genes related to adaptation to harsh environments, such as drought, acid soil, disease [21], and insect resistance, are present in wild oats and can have a direct or indirect impact on crop output and quality. As a result, crop polyploidization is critical for next-generation crop development in order to address problems with food security [20].

Understanding plant genomics is critical for breeding, conservation management, and genetic resource usage. Modern breeding methods use markers to choose safe and extremely advantageous variations, resulting in consistent quality implantation from wild relatives and improved varieties [[22], [23], [24], [25]]. Morphological markers, such as flower color and seed shape, are essential for assessing hereditary differences in improved varieties [24,[26], [27], [28]]. Isozymes and allozymes are biochemical markers that qualitatively discriminate distinct phenotypes and are used to evaluate heritable features and plant genetics management [29]. Various methods are used, each having pros and cons: isomers, inter-simple sequence repeats (ISSRs), restriction fragment length polymorphisms (RFLPs), random amplified polymorphic DNAs (RAPDs), and simple sequence repeat (SSR) markers. SSRs are DNA sequences found in eukaryotic genomes and are faster and more cost-effective than RFLP and AFLP markers [[30], [31], [32]].

Genetic diversity, also known as genetic divergence or genetic distance, refers to the heritable variation within and across populations of a species. It is critical for agricultural improvement activities since it aids in identifying parental combinations, introducing desired genes, and categorizing accessions for specific breeding goals. Accurate measurement of genetic diversity is critical to breeding success [33]. Its huge genome size, several linkage groups, and high ploidy levels make analyzing the hexaploid oat genome more difficult [34]. These cause a typical complexity for identifying multilocus linkage map by one probe due to the migration of fragments of diverse loci. Furthermore, they make it difficult to evaluate allele connections and conduct genetic analysis [35].

Exploratory the genetic diversity and population structure of Ethiopian oats using molecular markers has not received much attention. This made it reasonable to use SSR markers to investigate the genetic diversity and population structure of Ethiopian oat accessions in order to accomplish the following specific objectives: 1) Determine the magnitude of genetic diversity in local and exotic oat species using SSR markers; and 2) Determine the magnitude of genetic diversity between wild and cultivated oat accessions using SSR markers.

## 2 Materials and methods

### 2.1 Experimental materials

**Fig. 1:**
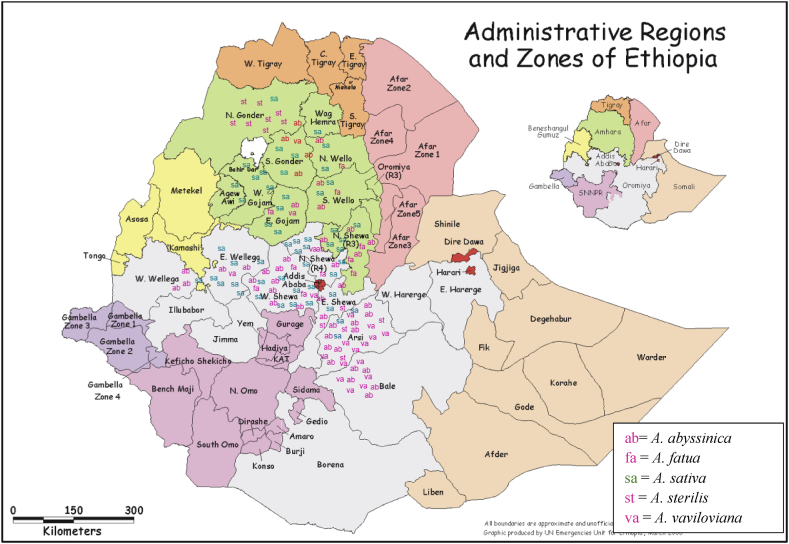
The geographical distribution of 148 Ethiopian oat accessions Arsi, Bale, Gojam (Agew Awi, E. Gojam, and W. Gojam zones), Gondar (N. Gondar and S. Gondar zones), Shewa (N. Shewa, E. Shewa, and W. Shewa zones), Wellega (E. Wellega and W. Wellega zones), and Wello (N. Wello, S. Wello, and Waghemra zones). The map was composed by Tamene [36].

The samples were gathered from the stem with the floret, and the 116 accessions were identified by experts at the Addis Ababa University Herbarium. Furthermore, the Ethiopian Biodiversity Institute (EBI) provided 34 accessions of *Avena* species, 32 from Ethiopia and two from the Netherlands. The International Livestock Research Institute (ILRI) in Addis Ababa supplied an extra 26 A. *sativa* accessions. The ILRI received 23 submissions from the USA and three from Australia. Ethiopia had 148 accessions out of a total of 176, the USA had 23, and Australia and the Netherlands jointly had five accessions. Ethiopian accessions were gathered from the provinces of Gojam, Gondar, Wello, Shewa, Arsi, Bale, and Wellega, which are located in the two regions (Amhara and Oromia region) where oats were fairly cultivated (Fig. 1). The 176 accessions were divided into five species: *A. sativa* had 82, *A. fatua* had 18, *A. sterilis* had 12, *A. abyssinica* had 44, and *A. vaviloviana* had 20. *A. sativa* L. is the cultivated variety, and the wild species include *A. abyssinica, A. vaviloviana*, *A. fatua*, and *A. sterilis*.

### 2.2 Establishment of **plant materials and DNA extraction**

The healthy leaf tissues that were collected from the Addis Ababa University greenhouse in 2018 were dried using silica gel. The genomic DNA of randomly selected dry leaves was extracted with the Quick-DNA Plant/Miniprep extraction kit protocol.

### 2.3 Quantity and quality of DNA

DNA sample the quantity and purity are measured and assessed using gel-based techniques and nanodrop spectrophotometry. The quantity and quality of the DNA samples were evaluated using Nanodrop using the standard spectral pattern for DNA, the A260/A280 ratio. A 100-bp λ-DNA marker was applied to a 0.8 % agarose gel for 1 h at 80 V in Agaros gel in order to measure the quantity, purity, and integrity of DNA. The PCR process used 20 ng/L of diluted DNA material.

### 2.4 PCR primers and amplification

Based on an earlier study, SSR markers with high polymorphism content were chosen. A preliminary batch of 19 SSR primer pairs was employed. They were developed by Li [37] and Pal [38] for *Avena*, by Gao [39] and Gao [40] for wheat, and by Liu [41] and Russell [42] for barley. The markers' colors (dye), repeat motifs, and sequences are displayed in Table 1.

**Table 1:** 4. A list of the 19 SSR primers that were used to analyze the polymorphism in 176 oat accessions that were gathered from Ethiopia, the United States, Austria, and the Netherlands.

SSR Marker	F - Primer 5' to 3' R - Primer 5' to 3'	Motif	Ta (°C)	Major Allele Freq	Number Allele	G D	PIC	Color of F - Primer
AM3	F_CTGGTCATCCTCGCCGTTCA R_CATTTAGCCAGGTTGCCAGGTC	(AG)_35_	55	25.8 %	13	88.1 %	87.3 %	NED
AM4	F_GGTAAGGTTTCGAAGAGCAAAG R_GGGCTATATCCATCCCTCAC	(AG)_34_	58	9.7 %	21	95.8 %	95.7 %	NED
AM6	F_AATGAAGAAACGGGTGAGGAAGTG R_CCAGCCCAGTAGTTAGCCCATCT	(AG)_20_	58	34.4 %	9	76.2 %	73.0 %	6-FAM
AM7	F_GTGAGCGCCGAATACATA R_TTGGCTAGCTGCTTGAAACT	(AG)_21_	58	17.7 %	28	95.2 %	95.0 %	PET
AM14	F_GTGGTGGGCACGGTATCA R_TTGGCTAGCTGCTTGAAACT	(AC)_21_	65	26.3 %	12	87.2 %	86.2 %	6-FAM
AM15	F_GTGACCGTAAACGATAACAAC R_AAGCAAGACGCGAGAGTAGG	(AC)_14_	55	29.6 %	11	86.9 %	86.0 %	VIC
AM21	F_ACGTTGGTCTCGGGTTGG R_TTGGCTAGCTGCTTGAAACT	(AT)_5_.(AC)_5_.(AC)_5_	55	38.7 %	8	75.1 %	71.9 %	NED
AM22	F_ATTGTATTTGTAGCCCCCAGTTC R_AAGAGCGACCCAGTTGTATG	(AC)_22_	65	24.7 %	23	93.0 %	92.8 %	PET
AM23	F_TCTTTAAGGATTTGGGTGGAG R_AAGAGCGACCCAGTTGTATG	(AC)_19_	65	52.7 %	5	63.7 %	58.8 %	6-FAM
AM30	F_TGAAGATAGCCATGAGGAAC R_GTGCAAATTGAGTTTCACG	(GAA)_14_	55	16.1 %	15	94.1 %	93.8 %	VIC
AM31	F_GCAAAGGCCATATGGTGAGAA R_CATAGGTTTGCCATTCGTGGT	(GAA)_23_	59	5.9 %	28	98.5 %	98.4 %	NED
AM42	F_GCTTCCCGCAAATCATCAT R_CATAGGTTTGCCATTCGTGGT	(GAA)_16_	57	16.1 %	16	94.6 %	94.4 %	6-FAM
AM53	F_TCGCCATTAATAAGAGGGAAGG R_GCTGCTGTTGGGTGGTTAGTG	(AC)_10_	59	37.6 %	5	73.1 %	68.7 %	VIC
AM87	F_GAGCAAGCTCTGGATGGAAA R_CCCGTTTATGTGGTTGTTAGC	(AC)_13_	64	30.1 %	7	84.2 %	82.7 %	6-FAM
AM89	F_GGCGGTTGGAGAGTGTCT R_AGGTGAAGGCGAGTGGAAG	(AC)_16_	64	18.8 %	16	94.8 %	94.6 %	VIC
AM102	F_TGGTCAGCAAGCATCACAAT R_TGTGCATGCATCTGTGCTTA	(AC)_3_(AG)_4_	64	21.0 %	20	92.5 %	92.1 %	PET
CWM48	F_GATCGGCGACTTCCTCCCTCAT R_ACCCCGCTCTTTCCCCAATAAT	(ATG)_7_	65	11.8 %	28	97.3 %	97.2 %	NED
CWM204	F_GCTACAAACCAGTCAGCAA R_AACCGACCCTCCTCCTTC	(ATGT)_6_	54	24.2 %	7	87.9 %	86.9 %	VIC
HVMwG	F_TCCAATGGCATCTACAGGACGGCCAA R_GCAGGTTGAGCTGCGCAAAGTCGTCG	(AT)_9_	57	15.6 %	27	95.1 %	94.9 %	PET

**NB**. The microsatellites are abbreviated as ‘AM’ for *Avena,* CWM for wheat and HVMwG for barley. We took 16, 2, and 1 microsatellite for *Avena,* barley, and wheat, respectively.

The current investigation employed unlabeled reverse primers and 5' end-labeled forward primers of PET (red), 6-FAM (blue), VIC (green), and NED (yellow) (Table 1). A 10 mL total reaction volume was used for the PCR, which was conducted using an Accupower PCR premix tube. The package consists of 200 μM dNTPs, 10 mM Tris-HCl (pH 9.0), 2.0–2.5 mM MgCl2, lyophilized 1U Taq DNA polymerase, and a tracking and stabilizing dye (Bioneer, Daejeon, North Korea). Add 2.0–2.5 mL of 20 ng/μL DNA template, 2.0–2.6 mL of double distilled water, and 0.2 mL of 0.5 pM primers to the premix. Initial denaturation at 95 °C for 3 min is part of the PCR process. Thereafter, there are 35 cycles of denaturation at 95 °C for 30 s, annealing at 54–65 °C for 1 min, extension at 72 °C for 1 min, and final extension at 72 °C for 20 min.

### 2.5 Pooling of PCR products and SSR fragment analysis

Each amplified product was diluted in 0.8–1.4 mL of double-distilled water to create a pool of PCR products. The relative fluorescence unit on the ABI 3730 sequencer and the relative intensities of amplification resolved on a 0.8 % TBE agarose gel were used to calculate the dilution. By thoroughly combining 1000 mL of Hi-Di Formamide with 12.0 mL of 500 LIZ size standards, a standard cocktail mix for fragment analysis was produced. Next, mix 9.0 mL of cocktail mix with 1.0 mL of each pooled PCR result in a 96-well PCR plate. For 2 min, the PCR plates were spun down at 3000 rpm after being gently vortexed. After being denatured for 3 min at 95 °C, the mixture was quickly cooled on ice for 5 min, and then it was put into the ABI 3730 sequencer for fragment analysis.

For sizing, the Applied Biosystems Genemapper application V.4.1 was utilized, which employed reference data to carry out fragment size matching and peak identification. Allele calls were automatically made when a peak in a data sample matched a bin's location. Completed results were run through AlleloBin software [43] to fix any mistakes in the scored alleles. The errors were caused by DNA polymerase slippage during PCR, which results in stuttering peaks [44]. Next, the ALS-Binary program was used to convert the allele calls into binary data [45] for the subsequent analysis.

### 2.6 Data analysis

The genetic diversity was evaluated using GenAlex version 6.5 [46]. Using the Nei and Li [47] methodology, the total number of alleles per locus (Na), effective number of alleles per locus (Ne), observed heterozygozity (Ho), Shannon's information index (I), and gene diversity (He) were all calculated. For every SSR locus, PIC values were calculated using the formula PIC = 1-Σ (pi2), where pi represents the ith allele frequency. To ascertain the level of variety within and across the sources of collection of the oat accessions, an analysis of molecular variance (AMOVA) was conducted. In STRUCTURE version 2.3.4, the Bayesian clustering approach was used to identify the population structure of the 176 oat accessions [48]. Markov Chain Monte Carlo (MCMC) and the burn-in period were both set to 10,000 iterations [49]. Twenty runs were carried out for each K-value (assumed number of subpopulations) ranging from 1 to 10 in order to provide an accurate estimate of the number of populations. Additionally, the Evanno et al. [49] approach was used with the STRUCTURE Harvester program [50] to estimate the optimal K value and determine Delta K values. The 176 genotypes were grouped according to their genetic relationships, or relatedness, using the genetic dissimilarity coefficients. The dendrogram was created using the unweighted pair group method (UPGMA) in DARwin 6.0 [50].

## 3 Results

### 3.1 Genetic variation

#### 3.1.1 Genetic variability across 19 SSR markers

All markers have PIC values larger than 50 %, ranging from 58.8 % to 98.4 %, and are regarded as useful markers for exploring genetic variation. At marker AM31, the PIC value was highest, while at marker AM23, it was lowest. Except for CWM204, AM21, and AM102, which have distinct repeating motifs, the majority of core SSR sequence repeat motifs used in this study are di- and trinucleotides (Table 1). The main allele frequency ranged from 5.9 % in AM31 to 52.5 % in AM23.

#### 3.1.2 Based on the place of collection, the genetic relationships between 176 oat accessions

Based on the source of collection, Table 2 displays the genetic heterogeneity among oats. The observed (Na) and effective (Ne) numbers of recognized alleles had average values of 3.85 and 2.74, respectively. The Netherlands and Australia had the lowest Na and Ne, at 1.93 and 1.76, respectively. Similarly, Shewa collections had the greatest Na and Ne values (5.67 and 3.26, respectively). Across all oat genotypes, the average observed Ho and He values were 0.69 and 0.48, respectively. Oat accessions from Bale, as well as the combined oat accessions from the Netherlands and Australia, have the lowest values of Ho (0.60) and He (0.37). The greatest values of Ho = 0.78 and He = 0.58 were displayed by Shewa (Table 2).

**Table 2:** The 176 oat accessions' genetic diversity parameter, categorized by collection areas.

Pop	Parameter				
	Na	Ne	I	Ho	He
Arsi	5.09	3.42	1.14	0.75	0.54
Bale	2.87	2.31	0.80	0.60	0.41
Gojam	3.07	2.35	0.80	0.62	0.42
Gondar	4.48	3.29	1.11	0.78	0.55
Netherlands and Australia	1.93	1.76	0.63	0.58	0.37
Shewa	6.78	3.85	1.28	0.78	0.58
USA	4.07	2.83	1.01	0.75	0.51
Wellega	3.48	2.52	0.92	0.64	0.46
Wello	2.87	2.38	0.82	0.67	0.44
Mean	3.85	2.74	0.94	0.69	0.48
SE	0.20	0.13	0.04	0.02	0.02

Notes: I is Shannon's information index; Na is the total number of alleles per locus; Ne is the number of effective alleles per locus; Ho is variability in genes seen among accessions; He is the mean genetic diversity among accessions. SE stands for standard error.

#### 3.1.3 Molecular variance

AMOVA results revealed significant genetic differences (P < 0.001) among populations, individuals, and within individuals (Table 4). Individuals among populations accounted for 18 % of the variance, while genetic variation among populations explained 4 %. Individual differences accounted for the remaining 78 % of the variance.

This suggests that genomes vary moderately within individual populations rather than between individuals and populations. The FST value of 0.039 shows moderate differentiation between locations, with a significant deviation from zero (P < 0.001). AMOVA analysis of *Avena* species revealed significant variability, with 33 % variation among the five species and 67 % within each species, showing considerable species distinctiveness.

### 3.2 Genetic distance and gene differentiation

#### 3.2.1 Genetic distance and genetic differentiation among the population of 176 accessions

Nei's unbiased genetic distance was employed in the investigation [51]. The accessions obtained in Bale and the Netherlands and Australia together had the greatest genetic distance (1.92) according to the average Nei's unbiased genetic distance. Arsi and Gondar accessions had the lowest genetic distance (0.97). In terms of genetic identity, they ranged from 0.15 to 0.38 (Table 3). Arsi and Gondar accessions shared the highest genetic identity (0.38).

**Table 3:** The molecular variance analysis both within and among the 176 oat accessions.

Source variation	df	SS	MS	Estimated variance	Percent variation	Fst	P – value
Among Pops	8	5455003.7	681875.5	10416.2	4 %	0.039	0.001
Among Individuals	167	50816287.8	304289.1	47400.4	18 %		
Within Individuals	176	36869961.0	209488.4	209488.4	78 %		
Total	351	93141252.5		267305.0	100 %		

Noted: Df = degree of freedom, SS = , MS = .

**Table 4:** Pairwise estimations for genetic differentiation (above diagonal off brackets), gene flow (above diagonal within brackets), genetic distance (below diagonal off brackets), and genetic identity (below diagonal within brackets).

Gene differentiation and Gene flow									
	Arsi	Bale	Gojam	Gondar	NAA	Shewa	USA	Wellega	Wello
Arsi		0.37(0.43)	0.38(0.41)	0.29 (0.61)	0.41 (0.36)	0.32 (0.53)	0.32 (0.53)	0.37 (0.43)	0.36 (0.44)
Bale	1.17(0.31)		0.46 (0.29)	0.38 (0.41)	**0.50 (0.25)**	0.36 (0.44)	0.41 (0.36)	0.45 (0.31)	0.43 (0.33)
Gojam	1.35(0.26)	1.58(0.21)		0.37 (0.43)	0.46 (0.29)	0.36 (0.44)	0.38 (0.41)	0.44 (0.32)	0.420 (0.35)
Gondar	**0.97(0.38)**	1.28(0.28)	1.25(0.29)		0.41 (0.36)	**0.28 (0.64)**	0.32 (0.53)	0.36 (0.44)	0.35 (0.46)
NAA	1.44(0.24)	**1.92(0.15)**	1.36(0.26)	1.39(0.25)		0.39(0.39)	0.41 (0.36)	0.45 (0.31)	0.45 (0.31)
Shewa	1.00(0.37)	1.22(0.30)	1.32(0.27)	0.98(0.37)	1.40(0.25)		0.30 (0.58)	0.35 (0.46)	0.33 (0.51)
USA	1.15(0.32)	1.61(0.20)	1.22(0.30)	1.13(0.32)	1.27(0.28)	1.15(0.32)		0.37 (0.43)	0.35 (0.46)
Wellega	1.45(0.23)	1.69(0.19)	1.61(0.20)	1.35(0.26)	1.43(0.24)	1.28(0.28)	1.28(0.28)		0.40 (0.38)
Wello	1.22(0.29)	1.43(0.24)	1.33(0.26)	1.04(0.35)	1.41(0.24)	0.99(0.37)	1.06(0.35)	1.25(0.29)	
Genetic distance and Genetic identity									

Note: NAA- Netherlands and Australia collections.

Genetic differentiation ranged from a low of 0.28 between the Gondar and Shewa collections to a high of 0.50 between jointly the Netherlands and Australia and Bale collections. Overall, our findings revealed significant genetic differentiation amongst groups. Gene flow (Nm) varied from low (0.25) between the Netherlands and Australia with Bale accessions to high (0.64) between the Shewa and Gondar collections (Table 3). In general, limited gene flow was seen between populations due to their high genetic divergence.

#### 3.2.2 Genetic distance and gene differentiation among five *Avena* species

*A. sterilis* and *A. sativa* had an unbiased genetic distance of 0.51, while *A. vaviloviana* and *A. abyssinica* had an unbiased genetic distance of 1.47. The least gene differentiation (0.20) was discovered between *A. sterilis* and *A. vaviloviana*, whereas the highest gene differentiation (0.39) was discovered between *A. abyssinica* and *A. vaviloviana*. In terms of gene differentiation, *A. sativa* accessions had the highest gene differentiation from *A. vaviloviana* followed by *A. fatua*, *A. abyssinica*, and *A. sterilis*, respectively (Table 5). The range of the gene flow was 0.39–1.03.

**Table 5:** The pairwise population matrix displays the Nei unbiased genetic distance (above diagonal off brackets), Nei unbiased genetic identity (above diagonal within brackets), genetic differentiation (below diagonal off brackets), and gene flow (below diagonal inside brackets) of the five *Avena* species.

Nei unbiased genetic identity and Nei unbiased genetic distance					
A.*abyssinica*	A.*fatua*	A.*sativa*	A.*sterilis*	A.vavilovina	
	0.88(0.41)	0.80(0.45)	0.92(0.40)	**1.47(0.23)**	A.*abyssinica*
0.29(0.61)		0.84(0.43)	0.79(0.45)	1.41(0.24)	*A.fatua*
0.24(0.81)	0.23(0.85)		**0.51(0.60)**	1.14(0.32)	*A.sativa*
0.30(0.59)	0.28(0.65)	**0.20(1.03)**		1.16(0.32)	*A.sterilis*
**0.39(0.39)**	0.38(0.41)	0.30(0.58)	0.36(0.45)		*A. vaviloviana*
genetic differentiation and gene flow					

#### 3.2.3 Cluster analysis of five *Avena species*

**Fig. 2:**
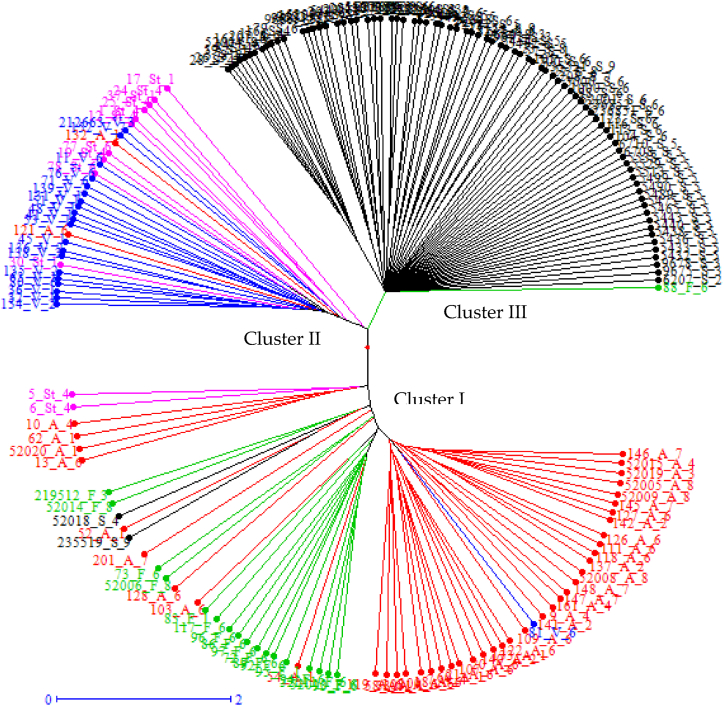
A radial tree analysis using 19 SSR markers identified a genetic relationship between 176 oat accessions of five *Avena* species^1^collected from different parts of Ethiopia, USA, the Netherlands and Austria.

**Fig. 3:**
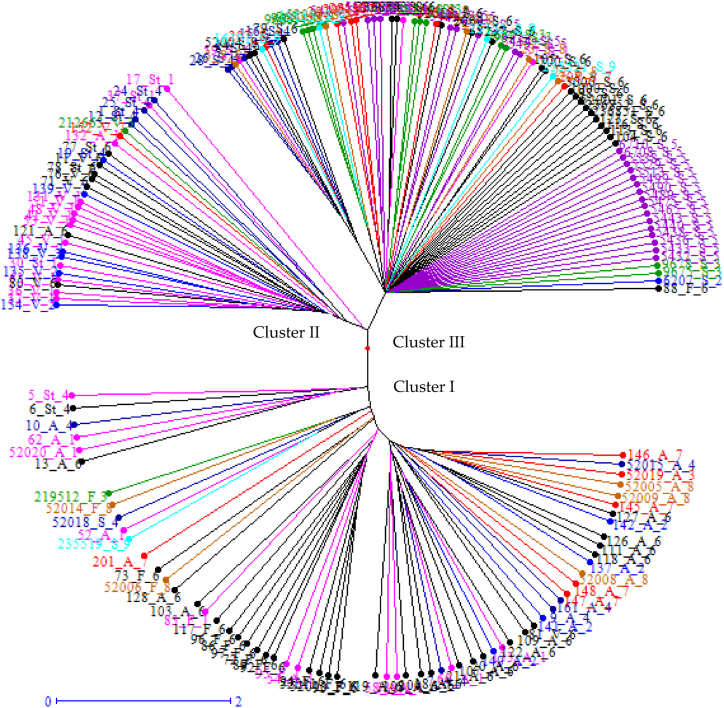
Details of clusters produced by 19 SSR markers in 176 oat accessions collected from different region of Ethiopia, USA, and the compiled of the Netherlands and Austria 2

Clusters were created based on species (Fig. 2) and geographical location (Fig. 3). The accessions were grouped into three clusters based on species. Cluster I contained the majority of *A. abyssinica (red)*, *A. fatua (green)*, accessions, two *A. sterilis* (pink), two *A. sativa* (black) accessions, and one *A. vaviloviana* (blue) accession (Fig. 2). Cluster II contains the majority of *A. sterilis* accession, *A. vaviloviana* accession, two *A. abyssinica* accessions, and one *A. sterilis* accession. Cluster III contains the majority of *A. sativa* accessions, with the exception of one *A. fatua* accession.

Geographical location, accessions from Ethiopia were more evenly distributed among the three clusters, regardless of the species type, whereas accessions from the USA were concentrated in Cluster III alone (Fig, 3 and Table 6).

**Table 6:** Description of the oat accessions selected from the clusters derived from the dendrogram. There were a total of 176 accessions falling under different clusters, cluster I to luster III.

Cluster name	Geographical location name	Membership no.
Cluster I	Arsi, Bale, Gojam, Gondar, Shewa, Wellega, Wello, the Netherlands and Australia.	66
Cluster II	Arsi, Bale, Gojam, Gondar, Shewa, Wellega, Wello	32
Cluster III	Arsi, Bale, Gojam, Gondar, USA, Shewa, Wellega, Wello, the Netherlands and Australia.	78

#### 3.2.4 Principal coordinate analysis (PCoA) for five *Avena* species

**Fig. 4:**
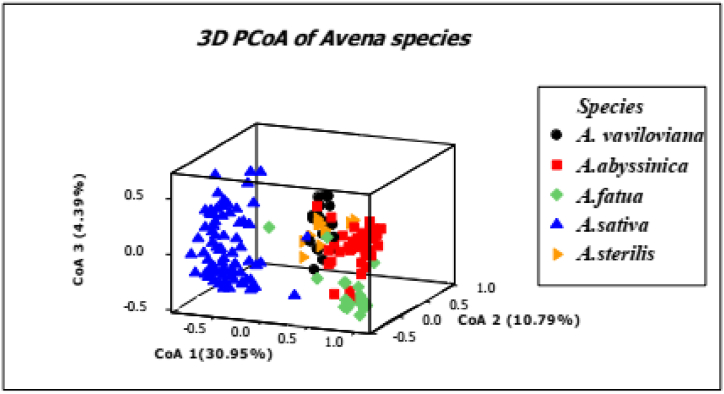
The ranges of 176 oat accessions from five different *Avena* species are displayed in a PCO scatter diagram analysis. First, second, and third coordinates are the major coordinates, in that order.

To comprehend the diversity and structure of oat accessions, principal coordinate analysis (PCoA) was used (Fig. 4). Of the SSR variation, 46.13 % could be explained by the first three main components. The accessions assigned by GenAlex 6.5 to the first and second clusters were divided by the first axis, which explained 30.95 % of the variation [46]. The most varied group of landraces from the third cluster was formed by the second axis, which explained 10.79 % of the variance.

The majority of the accessions are categorized according to species type rather than originating region (Fig. 4). Of the overall variation, 41.74 % could be seen in the first two coordinates. *A. sativa* was found in cluster I of the PCOA, *A. sterilis* and *A.vaviloviana*, *A.abyssinica* and *A. fatua* in cluster II. Two significant distinct clusters were identified based on the accessions (I and II). Cluster II showed two subcluster, Subcluster I showed a cluster of *A.vaviloviana* and *A. sterilis*, whereas sub cluster II revealed a cluster of *A.abyssinica* and *A. fatua*.

#### 3.2.5 Population structure and differentiation

**Fig. 5:**
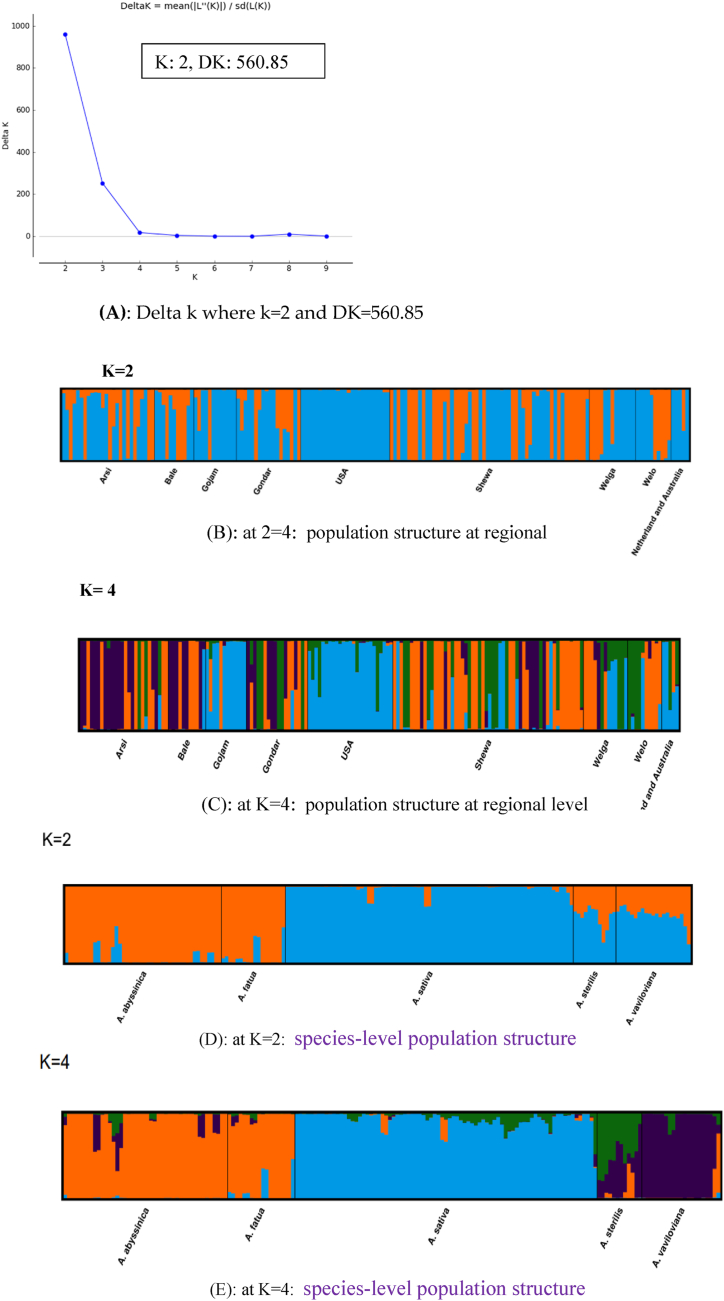
In (A), the number of unique genomic clusters (K) was displayed. Delta k where k = 2 and DK = 560.85 and Bayesian cluster analysis was done based on the geographical origin of the most optimal K = 2 and K = 4 clusters of **“B”** and **“C”** population structures of five *Avena* species, as well as **“D”** and **“E”** based on species type population structures of five *Avena* species at k = 2 and k = 4, respectively^3^

The parameters used to determine the number of subpopulations of oat accessions are the locations of a peak in the k distribution and a break point in the L (k) curve. Values of k = 2 (Fig. 5A) and k = 4 are supported (data not shown). The bulk of accessions were allocated by STRUCTURE to a subpopulation for both k values. It was able to discriminate between the cultivated oats and the wild accessions with k = 2 (Fig. 5**D**).

Consequently, it was possible to effectively divide the nine regional populations of oat accessions into two groups (subpopulations) (K: 2, DK: 560.85) (Fig. 5A). Fig. 5B and C depict the first group (blue subpopulation) in the USA, the Netherlands, and Australia, and the second (red) subpopulation in Ethiopian accessions.

Additionally, the admixture of the two subpopulations was evident in the Ethiopian accessions. Similarly, with k = 2, accessions of wild and cultivated oats were divided according to species type (Fig. 5D). Based on k = 4 (Fig. 5E), the gene pools of each species varied. Although the three gene pools in *A. sativa* were not equally distributed, this species possessed a richer gene pool than other species. Three genepools were present in *A. sterilis*, two of which were shared by *A. sativa* and *A. vaviloviana*. A lesser amount of the third was shared by all the species. *A. sterilis* shared gene pool types were more prevalent among the three gene pools of *A. vaviloviana*. In contrast to the other oat species examined here, *A. fatua* and *A. abssinica* shared significantly more similar gene pools. Cluster analysis and PCoA (Fig. 4) further confirmed the outcome. In terms of population, the USA population at K = 4 (Fig. 5B) displayed two gene pools, while Gojam, the Netherlands, and Australia collectively displayed three gene pools. These could be the three groups with nearly exclusively *A. sativa* accessions. Similar gene pools were shared by Arsi, Bale, Shewa, and Gondar, among other population. Even though all populations had no equal sample size, the Shewa population had an almost equal gene pool proportion to the other populations (Fig. 5B).

## 4 Discussion

For different diversity studies, the information gathered from the accessions of these nine populations was utilized. While accession from the Netherlands and Australia were pooled and treated as a single population, those from the USA were considered a single population. Ethiopian accessions were divided into seven regions or populations.

A crucial step in oat breeding is assessing the genetic diversity of oat accessions. Finding parent lines and constructing cross combinations that work well for particular breeding purposes is made easier by the assessment results. The goal of the study was to identify appropriate parents for hybridization by assessing the genetic diversity and population structure among oat accessions. The 176 oat accessions that were gathered from three separate sources were examined genetically using 19 microsatellite markers in this study. The genetic improvement of yield and other economically significant traits in crops is determined by the genetic diversity present within crop species. The oat genotypes found in this study are an invaluable resource for expanding the genetic base and making quick progress in Ethiopian oat breeding. Numerous physical features can result from a high level of genetic variety.

The results of the genetic diversity study have important ramifications for the conservation and continuous use of Ethiopia's genetic resources in the production of oats.

This paper offers a thorough analysis of the genetic diversity of Ethiopian wild and cultivated species, since Ethiopia is the origin and variety of oat species. In order to preserve the greatest amount of genetic variety, ex situ and in situ conservation strategies might be aided by identifying distinct genetic groups and clusters with rare or unique alleles. It is possible to use the gene pool for future use by prioritizing acquisition and preservation of different wild oat species. To protect improved oat production from new diseases, pests and environmental pressures, conservation of diverse oat genetic resources is critical.

Genetic diversity information can select desirable traits from wild relatives and lead to oat breeding initiatives. By exploiting the unexplored diversity found in wild oat species, cultivated oats can have a large genetic base, increasing their resilience, adaptability and agricultural performance. It is necessary to employ conservation and exploitation techniques in harmony to guarantee the resource's long-term availability for breeding and research in order to make sustainable use of oat genetic resources possible. Increasing oat production in Ethiopia can be facilitated by collaboration between gene banks, researchers and farmers in the evaluation, identification and cultivation of different oat germplasm.

Using 19 primers, 299 alleles were produced in 176 oat accessions, with an average of 15.73 alleles per locus. There were 3.85 distinct alleles on average, depending on the population or location. There were 2.74 effective identified numbers of alleles. In 125 *A. sativa* samples, Fu [52] found 125 alleles of 25 SSRs, In the 96 *A. sativa* genotypes, Choudhary [53] found 129 alleles totaling 40 primers, with an average of 3.22 alleles per locus. Furthermore, 66 *A. sativa* alleles from seven primers that were examined in 84 cultivars and landraces were reported by Nersting [54]. Using various genotypes may be the reason for the variation in the number of alleles found for each locus. With regard to alleles, markers such as AM7, AM31, and CWM48 had the greatest number, whereas markers such as AM23 and AM53 had the lowest number.

Primers with good discriminating power of SSR markers were indicated by most combinations with PICs over 0.7 and highly informative results. Cabral [55] revealed that 62 % of the SSRs investigated in *Avena strigosa* and *Avena barbata* of the *Avena* species, were polymorphic; Boczkowska and Tarczyk [56], similarly showed that 59.5 % of the SSRs evaluated in *A. sativa* were polymorphic among *Avena* species. Their findings did not perfectly match ours. Likewise, in opposition to what we discovered, earlier research revealed that applying 40 SSR markers to 96 genotypes of *A. sativa* resulted in PIC values that ranged from 0.06 to 0.75, with an average of 0.45 [57]. The variation may be related to intra- and interspecies differences in the genotype and type of SSR primer. The percentage of polymorphic components largely dictates how helpful a marker is. The numerous species under investigation are genetically diverse, as seen by increased levels of polymorphism. These polymorphic primers can be used in other molecular studies, including gene tagging, association mapping, and Marker Assisted Selection (MAS).

Averages of 71.58 % polymorphism were found across the origins of accessions; the accessions contained polymorphism ranging from 55.81 % (jointly, the Netherlands and Australia accessions) to 83.72 % (USA accessions). The breeding system of the species and ecological elements like latitude, altitude, temperature, moisture availability, and other soil-related factors might be responsible for population variation. The average value of Shannon's information index (I), which ranged from 0.63 to 1.28, was 0.94 (Table 4). This agrees with what Barzin [58] found, reporting an index of 0.82. Another indication of genetic variability in the oat germplasm used in the inquiry was the high value of Shannon's information index in the current study.

The estimate of geographical origin-specific proportion of variation and within-geographic origin variation are largely correlated, estimating the contribution of a geographical origin to total SSR variation. According to Hawtin [59], the specific selection pressures that exist in a particular habitat, along with historical production and selection histories, all influence the geographic distribution patterns of farmed crop species. As a result, in order to fully capture all allelic variation changes of a particular agricultural plant, diversity studies have welcomed the inclusion of genotypes taken from various geographic locales. There were no hotspots areas receiving particular focus for conservation, but breeders should have to use all germplasm for further selection in order to obtain all genetic resources with unique alleles. The plant breeder should give special consideration to accessions that occur in separate clusters for breeding programs.

AMOVA found significant genetic variation (P < 0.001) across participants. AMOVA results revealed the lowest genetic variance (8 %) among populations, considerable variation (18 %) among individuals, and 78 % of variance within individuals, indicating that the examined accessions have sufficient diversity for breeding. Previous studies on oat germplasm have shown variations on a similar pattern [58]. Findings from AMOVA suggest that the genetic diversity of the test genotypes will only be partially captured by a given source. Gene divergence in oats may be increased through massive crosses to support population growth, as evidenced by the abundance of variety both within and between populations.

A measure of the genetic divergence between two populations is called the genetic distance. In comparison to previously published values based on isozyme markers [60], RFLP markers [61], and AFLP markers [7], the total genetic distance found in this study was smaller. When everything was considered, the diversity observed in accessions indicated that there was a great deal of variation amongst the different species groups within the collection.

We hypothesized that species within tetraploid and hexaploid species with the same genome and ploidy levels would have close genetic distances. Tetraploid and hexaploid species, on the other hand, have significant genetic distances between them due to differences in genome and ploidy levels. In contrast, the results of this analysis showed that the tetraploid *A. abssinica* and hexaploid *A. fatua* plody levels of the two genomes had the shortest Nei unbiased genetic distance. Similarly, the hexaploid species *A. sterilis* and *A. vaviloviana* do not differ significantly in terms of genetic makeup. On the other hand, a significant degree of genetic differentiation was observed between the two tetraploid species, *A. vaviloviana* and *A. abyssinica*, and the remaining hexaploid species. Given that both the D genome, found in hexaploid species, and the B genome, found in tetraploid species, came from the A genome, the most likely explanation for this type of relationship is that the D and B genomes are derived from the A genome and yet share similar traits. Tetraploid and hexaploid oats are thus less distinct from one another than might be expected.

Populations in the USA showed a small degree of variation within and between species as well as across accessions within the region of origin, suggesting that oat accessions differ very little from one another geographically. These findings might be the consequence of farmer-to-farmer seed exchanges or because the original germplasm imported to many areas was comparable; wild oats, wheat, and barley were transported alongside cultivated varieties. It was possible to distinguish between the wild and cultivated species (*A. sativa*) based on the species-wise pattern shown by the cluster. It was discovered that species with different genomes clustered together, but material with similar genomes did not. This finding runs counter to Achleitner [62] finding that materials with similar genomes cluster together. There were not many differences between the *A. sativa* populations in the USA.

Our results are similar with previous publications [52,55,64] and provide credence to the theory that *A. sativa* was domesticated independently of its wild ancestors [63]. These reports also highlight the distinct differences between wild and cultivated species. Patterns of genetic diversity and geographic origins do not appear to be clearly correlated, according to research on a variety of cereals [65].

Gene flow analysis is a valuable tool for understanding the evolutionary relationships and histories of *Avena* species. It can measure gene flow, detect inbreeding and introgression, assess reproductive isolation, and indicate adaptive variation. In terms of genetic differentiation, 0.00–0.05 denotes small genetic differentiation, 0.05–0.15 suggests moderate genetic differentiation, 0.15–0.25 denotes substantial genetic differentiation, and more than 0.25 denotes extremely large genetic differentiation [66]. The resultant limited gene flow and strong genetic differentiation may be related to the reduced genetic diversity across the sources included in this study's collection. The three categories of gene flow were low (Nm < 1), moderate (Nm = 1), and high (Nm > 1) according to Morjan and Rieseberg [67]. High levels of gene flow may indicate hybridization events and introgression, while low levels indicate reproductive barriers. Because oats are an orphan crop, farmers and traders exchanged seeds infrequently. Combining gene flow analysis with other data, such as phylogenetic analyzes and cytogenetic data, provides general conclusions about the evolutionary significance and relationships among *Avena* species. This holistic approach is critical to understanding the complex history and diversity of *Avena* species.

Computing genealogies among *Avena* species allows researchers to identify evolutionary relationships, potential hybridization events, genetic diversity, and evolutionary ecological, ecological, or cytogenetic differences. This information, combined with other data, will help to understand diversity, hybridization history and variability within the *Avena* genus, taxonomic classification, germplasm conservation and agronomic trait development.

The overall SSR genotypes are quite predictive of the wild versus cultivated type, according to a PCoA analysis of the whole data matrix. There is some correlation between the germplasm's species type and clustering on the major axes. Furthermore, the species *A. sativa* and *A.abyssinica* did not group according to their place of origin. This finding verified that accessions are categorized using AFLP markers according to their geographic origin, as reported by Ref. [62]. Accessions from the Netherlands and Australia were clustered in the first cluster, showing a relatively moderate divergence; most USA accessions were assigned to a single cluster, which was followed by Gojam. Cluster admixture was high in Ethiopian collections such as Shewa, Arsi, Gondar, Wello, and Wellega. These regions' accession has not shown any differentiation.

Population structure analysis identified two subpopulations (Fig. 5), indicating that the analyzed oat genotypes share a restricted genetic foundation. Moreover, the examination of population structure revealed that the genotypes studied belonged to the same group, suggesting that crosses between genetically unrelated parents are necessary to create breeding populations.

The UPGMA cluster analysis based on genetic dissimilarity, which employed the neighbor-joining approach, separated the 176 oat accessions into three main groupings. The current investigation's clustering pattern showed variation in oat genotypes. Three clusters of 176 oat genotypes were identified by Ashenafi Alemu [68] using SSR markers. But the cluster patterns did not match the population structure that the collection region was expected to have. This could be because genotypes gathered from comparable locations share ancestors or are members of the same gene pool, according to Ashenafi Alemu [68]. In contrast, there could be significant gene flow caused by cross-pollination, potential chromosomal or gene mutations, and different ancestral sources for the genotypes of oats that were investigated that resulted in genetic diversity. In the present investigation, numerous clusters were formed from accessions of distinct species gathered from different regions, while accessions of the same species collected from different regions combined into a single cluster. These findings are consistent with previous research, which found that geographical distance did not influence genetic distance between genotypes [69]. According to Kumar [70], it is not appropriate to use geographic location to evaluate genetic diversity during genotype selection. This may have happened as a result of farmers and traders in the area exchanging genetic material. By using recycled seed as planting material, Ethiopian oat farmers are able to increase the genetic similarity between the crops they grow.

## 5 Conclusions

*A. sativa*, an important crop in Ethiopia, is a secondary oat diversification center due to its wide altitude range, temperature, rainfall, and different edaphic environments. The current investigation discovered reasonable variability among oat accessions that could be used for future breeding. According to the analysis, 176 oat accessions had the highest expected or unbiased expected heterozygosity and the greatest number of effective alleles, suggesting the possibility of gene introgression into other Ethiopian oat genotypes. According to the findings, 18 out of the 19 SSR markers that were chosen were highly polymorphic and could be used to identify the tested oat accessions. Regardless of the source of collection, the cluster analysis divided the 176 oat accessions into three major genetic groups. It is possible that accessions 88_f, 5_st, 6_st, 81_v, 52018_s, and 235519_s have a distinct genetic composition due to the remarkable genomic patterns and linkages they displayed. In oat breeding programs, these can provide sources of novel genes. Consequently, the knowledge gained will help Ethiopia and other east African nations produce more oats.

## Funding

The project has no funding.

## CRediT authorship contribution statement

**Ashenafi Alemu Tiruneh:** Writing – original draft, Software, Investigation, Data curation, Conceptualization. **Kassahun Tesfaye Geletu:** Supervision, Conceptualization. **Nasser k Yao:** Supervision, Conceptualization. **Kifle Dagne Weldegiorgis:** Validation, Supervision, Formal analysis, Conceptualization.

## Declaration of competing interest

The authors declare the following financial interests/personal relationships that could be considered competing interests: Ashenafi Alemu Tiruneh reports that the University of Gondar provided administrative support. The study is supported by Addis Abaa University, which provides supervisors.

## Footnotes

1 Where, Red stands for *A. abyssinica*, Blue for *A. vaviloviana*, Green for *A. fatua*, Pink for *A. strilis*, and Black for *A. sativa*.

2 Where, 1 stands for Arsi, 2 for Bale, 3 for Gojam, 4 for Gondar, 5 for the USA, 6 for Shewa, 7 for Wellega, 8 for the Wello, and 9 for the Netherlands and Australia.

3 (A) While accessions from the USA contain only one gene pool, all Ethiopian accessions exhibit gene pool admixture, as do those from the Netherlands and Australia. C) *A. abyssinica* and *A. fatua* have one gene pool that is shared by both species, whereas *A. sativa* has one gene pool that is distinct from those of the other two. Conversely, *A. vaviloviana* and *A. sterilis* contain a higher share of two gene pools.
